# Comparison of early postoperative recovery in patients undergoing elective colorectal surgery before and after ERAS® implementation—a single center three-armed cohort study

**DOI:** 10.1007/s00384-024-04770-0

**Published:** 2024-12-02

**Authors:** Katharina Knab, Leon Aurnhammer, Sylvia Büttner, Steffen Seyfried, Florian Herrle, Christoph Reissfelder, Georgi Vassilev, Julia Hardt

**Affiliations:** 1 Department of Surgery, Medical Faculty Mannheim, University Medical Center Mannheim, Heidelberg University, Theodor-Kutzer-Ufer 1-3, D-68167 Mannheim, Germany; 2 Department of Biometry and Statistics, Medical Faculty Mannheim, University Medical Center Mannheim, Heidelberg University, Theodor-Kutzer-Ufer 1-3, D-68167 Mannheim, Germany

**Keywords:** ERAS®, Quality of recovery, Colorectal surgery

## Abstract

**Purpose:**

This study examines the impact of enhanced recovery after surgery (ERAS®) on patient recovery after elective colorectal surgery. The innovative PostopQRS™ tool was used for the analysis of patient recovery.

**Methods:**

This single-center study compares three cohorts: two retrospective cohorts before (A) and after (B) ERAS® implementation and a prospective cohort post-ERAS® implementation (C) using PostopQRS™. The present study was prospectively registered in the German Register of Clinical Trials (DRKS00026903).

**Results:**

A total of 153 patients were included from June 2020 to February 2022. Significant differences were observed in bowel function, oral food intake, opioid use, and PONV (postoperative nausea and vomiting) occurrence. By the day of discharge, 98% in cohorts B and C had bowel movements or stoma output, compared to 66% in cohort A (*p* < 0.001). Solid food intake on POD1 was higher in cohorts B and C (*p* = 0.025), while opioid use was lower (*p* = 0.003 and *p* < 0.001). Cohort C showed 90% recovery on discharge.

**Conclusion:**

This study demonstrates improved early mobility, reduced need for opioids, a higher rate of patients with solid food intake on POD1, and earlier bowel movement as well as excellent recovery following the colorectal ERAS® implementation.

**Supplementary Information:**

The online version contains supplementary material available at 10.1007/s00384-024-04770-0.

## Introduction

The implementation of the Enhanced Recovery after Surgery (ERAS®) protocol in colorectal surgery, following the 2018 ERAS® guidelines, includes several evidence-based recommendations aimed at reducing perioperative stress to maintain hemostasis and improve postoperative recovery [1]. The purpose of this study was to analyze the changes in early patient recovery after the implementation of the colorectal ERAS® protocol at the University Medical Center Mannheim, Germany.

In addition to pre- and perioperative recommendations such as minimally invasive approaches, local and regional anesthesia, balanced fluid management, and early removal of drains and tubes, ERAS® includes guidelines for early postoperative mobilization and oral food intake, standardized pain medication, and prophylaxis for perioperative nausea and vomiting (PONV). All these items contribute to a reduction in the length of hospital stay, complications, and overall costs [2].

While adherence to the ERAS® protocol can only indirectly indicate better patient well-being and happiness, the analysis of quality of recovery as a complex multidimensional construct can only be defined by the patient based on subject findings. Emotional well-being and cognitive recovery after surgery should be equally considered goals of the ERAS® program regarding patient recovery. As an evaluated tool, the postoperative quality of patient recovery scale (PostopQRS™) can be used. The PostopQRS™ includes 24 questions objectifying different aspects including physiological, nociceptive, functional, cognitive, and emotional recovery [3]. The implementation of the PostopQRS™ as a tool for analysis in postoperative recovery for patients undergoing colorectal surgery has not yet been conclusively investigated. This study is therefore dedicated, among other things, to evaluating the use of PostopQRS™ in the setting of colorectal surgery.

We propose the implementation of the colorectal ERAS® protocol leads to an improved physiological and mental recovery of patients following colorectal surgery, which may be objectively measured using PostopQRS™. Additionally, we expect early mobilization and bowel movement, early oral food intake, improved pain management with less need for opioids, and a reduced PONV rate.

## Materials and methods

This single-center three-arm cohort study included patients undergoing elective colorectal surgery at our hospital between June 2020 and February 2022. The primary endpoint was to detect improvements in early patient recovery after the implementation of the ERAS® protocol. Patient recovery was assessed using parameters including pain intensity (VAS, visual analogue scale), patient mobility, opioid analgetic medication requirements, time to first bowel movement, oral food intake, and signs of PONV. The tolerance of oral food intake was defined according to Grass et al. as the ability to eat at least two normal meals of solid food without developing nausea and/or vomiting [4]. In practice, a patient had to be able to eat at least two-thirds of the meal offered or as much as he usually ate preoperatively. The data for these parameters was obtained from patient records. All patients who had received or were planned for elective colorectal surgery during the time were screened and, if suitable, included in this study after patient information and consent. Only patients 18 years of age or older were included in this study. Oxycodone was used as the standard opioid while metamizole and paracetamol are the most used non-opioid drugs in pain management.

The total cohort of the study comprised all patients who underwent elective colorectal surgery at the University Medical Center Mannheim between June 2020 and February 2022. The individual cohorts and the corresponding periods were defined as follows: The first cohort (cohort A) consisted of all consecutive patients who underwent surgery before the implementation of the ERAS® protocol between June 2020 and September 2020. Evaluation of this cohort was conducted retrospectively by analyzing patient file documentation. The second cohort (cohort B) comprised patients who underwent surgery between October 2020 and July 2021 following the implementation of the ERAS® protocol. Evaluation of this cohort was also conducted retrospectively through analysis of patient data and file documentation. The third cohort included patients who underwent surgery after the implementation of the ERAS® protocol between August 2021 and February 2022 and was analyzed prospectively. These patients were visited during the first 3 days postoperatively and also completed the postoperative Quality of Recovery Scale PostopQRS™ survey. The PostopQRS™ survey was completed online via the website (https://www.postopqrs.com/). We selected postoperative days 1–3 as the follow-up time points for the endpoints to be collected, since it is not uncommon for patients to be discharged as early as day 4 after elective colorectal surgery and uncomplicated postoperative course. The aim was to have follow-up data for all patients that were as complete as possible. To ensure this, the period was set to POD3, since most patients were hospitalized and could be interviewed without complications up to this point.

The data was processed through SAS Software (version 9.4, SAS Institute Inc, Cary, NC, USA), and statistical analysis was performed using chi-square correlations for qualitative parameters. If necessary, Fisher’s exact test was performed. Mean, standard deviation, median, and interquartile range (IQR) were calculated for quantitative parameters. Shapiro–Wilk test was applied to determine the distribution form of quantitative parameters; in addition, stem-and-leaf diagrams were used for graphical presentation. Skewed-distributed variables were analyzed using the Kruskal–Wallis test. Significance was assumed at *p* < 0.05. Data safety was guaranteed by pseudonymization of the patient data. Missing data was addressed and considered during the statistical analysis.

The present study has received a positive ethics vote from the local ethics committee (ethics committee II, Medical Faculty Mannheim, University of Heidelberg) and was prospectively registered in the German Register of Clinical Trials (DRKS), the German primary register recognized by the WHO (World Health Organization). The study information stored there can be accessed at the following URL: http://drks.de/search/en/trial/DRKS00026903.

## Results

### Baseline characteristics

A total of 153 patients were included in this study, accounting for 50 patients each in cohorts A and B and 53 patients in cohort C. The baseline characteristics are included in Table 1. The cohorts showed no significant differences in terms of age, gender, ASA (American Society of Anesthesiologists) score, or BMI (body mass index). At 37.7% (*n* = 20) cohort C included significantly more smokers than cohorts A (18%, *n* = 9) and B (6%, *n* = 3). Most patients had received prior abdominal surgery with no significant difference between the cohorts (49% (*n* = 24) in cohort A, 52% (*n* = 26) in cohort B, and 79.2% (*n* = 42) in cohort C). Surgical procedures included the full spectrum of colorectal surgery including right and left hemicolectomy, resection of the sigmoid, low anterior resection, and abdomino-perineal resection among others (stoma reversal, multivisceral resection, sub-/total colectomy, proctectomy with ileoanal pouch) with no significant differences between the three cohorts.

**Table 1:** Baseline characteristics and surgical approach, use of locoregional anesthesia by cohorts A, B, and C

	Cohort A (*n* = 50)	Cohort B (*n* = 50)	Cohort C (*n* = 53)	*p*
Age (years), median (IQR)	65.1 (52.5–71.7)	(64.7 (52.9–77.7)	65.8 (56.7–70.6)	0.718
Gender (female/male)	30/20	24/26	25/28	0.352
BMI (kg/m^2^), median (IQR)	24.1 (21.6–27.8)	26.4 (21.9–29.1)	21.6 (18.0–30.4)	0.377
Smoking (absolute number (%))	9 (18%)	3 (6%)	20 (37.7%)	** < 0.01**
ASA (absolute number (%))				0.431
1	8 (16.7%)	9 (18%)	4 (7.5%)	
2	30 (62.5%)	29 (58%)	32 (60.4%)	
3	10 (20.8%)	12 (24%)	17 (32.1%)	
Previous abdominal surgery (absolute number (%))	24 (49%)	26 (52%)	42 (79.3%)	**0.002**
Surgical approach (absolute number (%))				**0.004**
Laparoscopic	38 (76.0%)	43 (86.0%)	38 (71.7%)	
Open	12 (24%)	2 (4%)	12 (22.6%)	
Robotic-assisted	0 (0%)	5 (10%)	3 (5.7%)	
Locoregional anesthesia				
TAP block	12 (24%)	16 (32%)	27 (50.9%)	**0.014**
Epidural catheter	10 (21.3%)	3 (6%)	6 (12.5%)	0.083

Significant *p*-values in **bold**

*ASA* Physical Status Classification System by American Society of Anesthesiologists, *BMI* body mass index, *IQR* interquartile range; *TAP block* transversus abdominis plane block

Most patients received surgery laparoscopically, with 76% (*n* = 38) in cohort A, 86% (*n* = 43) in cohort B, and 72% (*n* = 38) in cohort C with no significant difference between the cohorts. Open surgery was performed in 24% (*n* = 12) of the cases in cohort A and 23% (*n* = 12) in cohort C, while only 4% (*n* = 2) in cohort B received open surgery. Robotic surgery was exclusively performed in cohorts B (10%, *n* = 5) and C (6%, *n* = 3). Fifty-one percent (*n* = 27) of the patients in the prospective ERAS® cohort (C) received a TAP (transversus abdominis plane) block intraoperatively, significantly more than in cohort A at 24% (*n* = 12) and in cohort B at 32% (*n* = 16) (*p* = 0.014).

Compliance regarding ERAS® items varied greatly in the ERAS® cohorts B and C depending on the perioperative phase: preoperative and intraoperative compliance were very high in both cohorts (ranging from 73 up to 82.9%), whereas pre-admission and postoperative compliance were noticeably lower (ranging from 33.1 up to 51.1%; for details, see supplementary material).

### Endpoints

The detected endpoints show significant differences between the cohorts for bowel function, oral food intake, opioid intake, paracetamol intake, and PONV. All data is presented in Table 2.

**Table 2:** Endpoints by cohorts and categories in absolute numbers and percentage

	Cohort A (*n* = 50)	Cohort B (*n* = 50)	Cohort C (*n* = 53)	*p*
Bowel function				
POD1	22 (44.9%)	27 (54%)	26 (49.1%)	0.663
POD2	31 (64.6%)	38 (76%)	40 (75.5%)	0.363
POD3	37 (82.2%)	34 (89.5%)	49 (98%)	**0.029**
Day of discharge	31 (66%)	49 (98%)	52 (98.1%)	** < 0.001**
Time to first bowel movement (days), median (IQR)	2 (1–2)	1 (1–2)	2 (1–2)	0.306
Oral feeding				
POD1	21 (42.9%)	35 (70%)	30 (56.6%)	**0.025**
POD2	36 (75%)	46 (92%)	46 (86.8%)	0.057
POD3	39 (86.7%)	37 (97.4%)	45 (90%)	0.224
Day of discharge	47 (100%)	49 (98%)	52 (98.1%)	1.000
Time to first tolerated oral normal diet (days), median (IQR)	2 (1–2)	1 (1–2)	1 (1–2)	**0.009**
Postoperative ileus rate, absolute number (percentage)	1 (2%)	5 (10%)	3 (5.7%)	0.243
Overall complications (number of patients with at least 1 complication)	8 (16%)	11 (22%)	15 (28.3%)	0.324
Complications (Clavien Dindo)				0.282
Grade 1	4 (8%)	9 (18%)	7 (13.2%)	
Grade 2	0	1 (2%)	1 (1.9%)	
Grade 3a	2 (4%)	0	1 (1.9%)	
Grade 3b	1 (2%)	1 (2%)	5 (9.4%)	
Grade 4a	1 (2%)	0	0	
Grade 4b	0	0	1 (1.9%)	
Grade 5	0	0	0	
Opioid intake				
POD1	16 (32%)	11 (22%)	15 (28.3%)	0.526
POD2	21 (42%)	6 (12%)	14 (26.4%)	**0.003**
POD3	19 (38%)	3 (6%)	14 (26.4%)	** < 0.001**
Day of discharge	7 (14%)	3 (6%)	3 (5.7%)	0.279
Paracetamol intake				
POD1	5 (10%)	16 (32%)	16 (30.2%)	**0.017**
POD2	5 (10%)	16 (32%)	12 (22.6%)	**0.027**
POD3	2 (4%)	10 (20%)	8 (15.1%)	0.052
Day of discharge	3 (6%)	10 (20%)	3 (5.7%)	**0.027**
Metamizole intake				
POD1	45 (90%)	47 (94%)	52 (98.1%)	0.194
POD2	47 (94%)	46 (92%)	49 (92.5%)	1.000
POD3	41 (82%)	35 (70%)	44 (83%)	0.208
Day of discharge	34 (68%)	40 (80%)	40 (75.5%)	0.380
PONV				
POD1	4 (8,5%)	10 (20%)	8 (15%)	0.277
POD2	3 (6.25%)	5 (10%)	11 (20.8%)	0.072
POD3	3 (6.7%)	5 (13%)	13 (26%)	**0.031**
Day of discharge	0 (0%)	0 (0%)	3 (5.7%)	0.108
Length of stay (days), median (IQR)	5 (4–9)	5 (4–7)	5 (4–7)	0.904

Significant *p*-values in **bold**

*IQR* interquartile range, *POD* postoperative days 1, 2, and 3, *PONV* postoperative nausea and vomiting

On POD1 and POD2, no significant difference was observed between the cohorts regarding bowel movement or stoma output. However, by POD3, almost all patients in cohorts B and C (89.5%, *n* = 34 and 98%, *n* = 49) had experienced bowel movement or stoma output, whereas in cohort A, this figure was significantly lower at 82.2%, *n* = 37 (*p* = 0.029). The difference on the day of discharge was even clearer: while in cohort A only 66% had a bowel movement, the rate in cohorts B and C was 98% (*p* < 0.001).

On POD1, in cohort A, 42.9% (*n* = 21) of the patients had consumed a solid regular diet. In contrast, significantly more patients in cohort B (70%, *n* = 35) and cohort C (56.6%, *n* = 30) had consumed solid food (*p* = 0.025). At POD2, 75% (*n* = 36) in cohort A consumed a solid regular diet. The percentage was higher in cohort B (92%, *n* = 46) and cohort C (86.8%, *n* = 46), although without statistical significance (*p* = 0.06).

In cohort A, the median time to the first tolerated intake of a normal oral diet was 2 days, significantly longer than in the ERAS® cohorts, where the median was 1 day in each case (0.009). The median time to first bowel movement was 2 days in cohorts A and C versus 1 day in cohort B (*p* = 0.306). There was also no significant difference regarding the median length of stay, which was 5 days in all cohorts (*p* = 0.904). Postoperative ileus occurred in one patient in cohort A (2%), while the incidence was 10% (5/50) and 5.7% (3/53) in the ERAS® cohorts, respectively (*p* = 0.243). Further details about these outcomes as well as complication rates are shown in Table 2.

As metamizole and if necessary paracetamol were used as the standard analgesic medication in our ERAS® program, metamizole was given in over 90% of patients in cohorts B and C during POD1 and POD2. Metamizole was taken equally in cohort A (90%, *n* = 45 POD1, 94%, *n* = 47 POD2, and 82%, *n* = 41 POD3) with no significant difference between the cohorts. Paracetamol intake was significantly higher in the ERAS® cohorts on POD1, POD2, and POD3. While in cohort A, paracetamol intake was at 10% (*n* = 5) or less, patients in cohorts B and C 32% (*n* = 16), and 30.2% (*n* = 16) of patients received paracetamol on POD1 (*p* = 0.017). On POD3, the intake was 20% (*n* = 10) in cohort B and 15.1% (*n* = 8) in cohort C.

On POD1, oxycodone intake was 32% (*n* = 16) in cohort A, with fewer patients needing oxycodone in cohorts B and C, at 22% (*n* = 11) and 28.3% (*n* = 15), respectively. However, the difference was not significant. On the following days, in POD2 and 3, the opioid intake of cohorts B and C was significantly lower (*p* = 0.003 and < 0.001).

Symptoms of PONV were reported in only 8.5% (*n* = 4) of patients in cohort A on POD1, but 20% (*n* = 10) and 15% (*n* = 8) in cohorts B and C with no significant difference between the cohorts (*p* = 0.277), respectively. On POD3, symptoms were reported in cohort C more often (26%, *n* = 13), whereas in cohort A, only 6.7% (*n* = 3) of patients suffered from nausea and vomiting (*p* = 0.031).

**Fig. 1:**
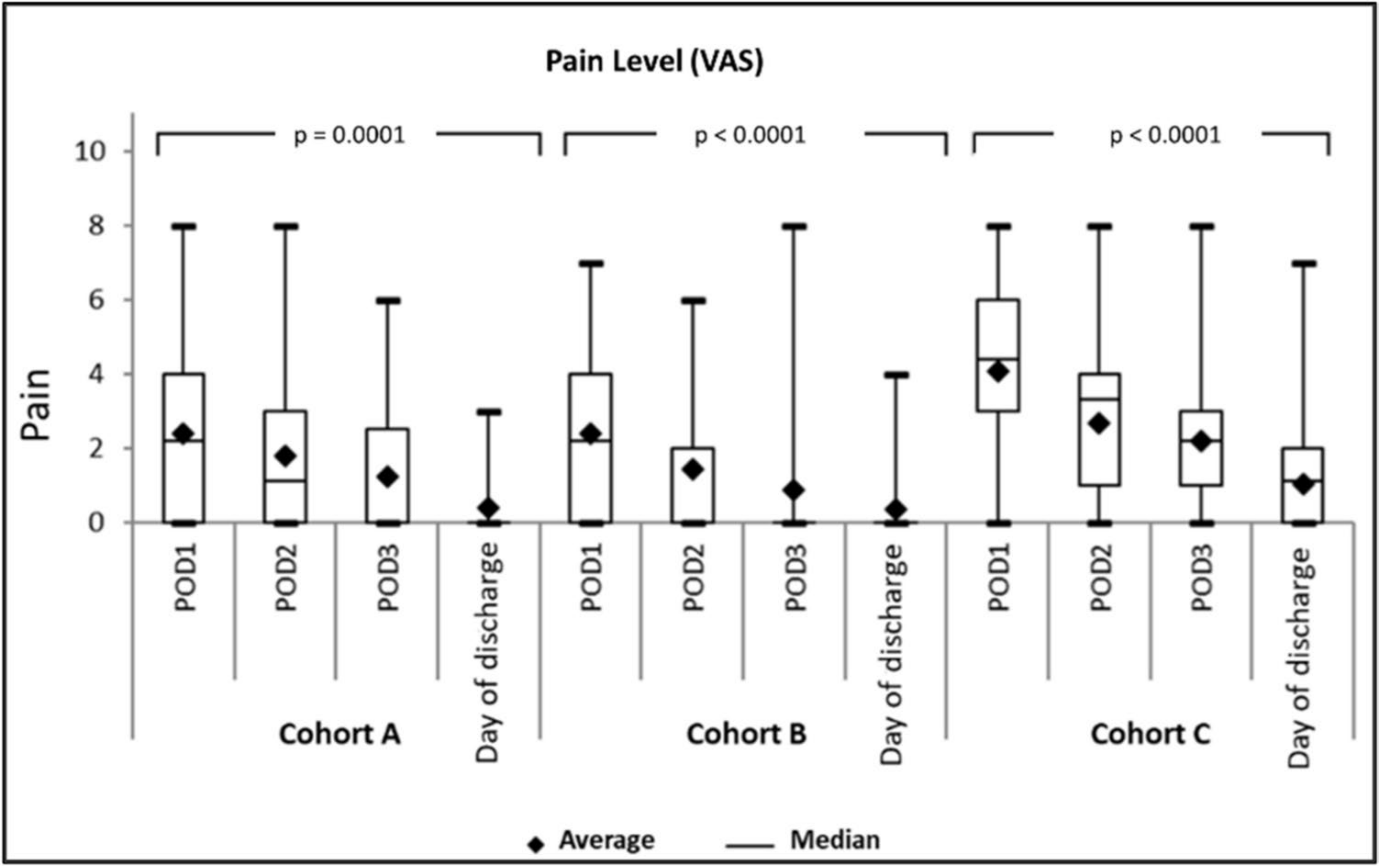
Boxplot cohort pain levels by VAS (visual analogue scale), average and median on postoperative days 1, 2, 3, and day of discharge

**Fig. 2:**
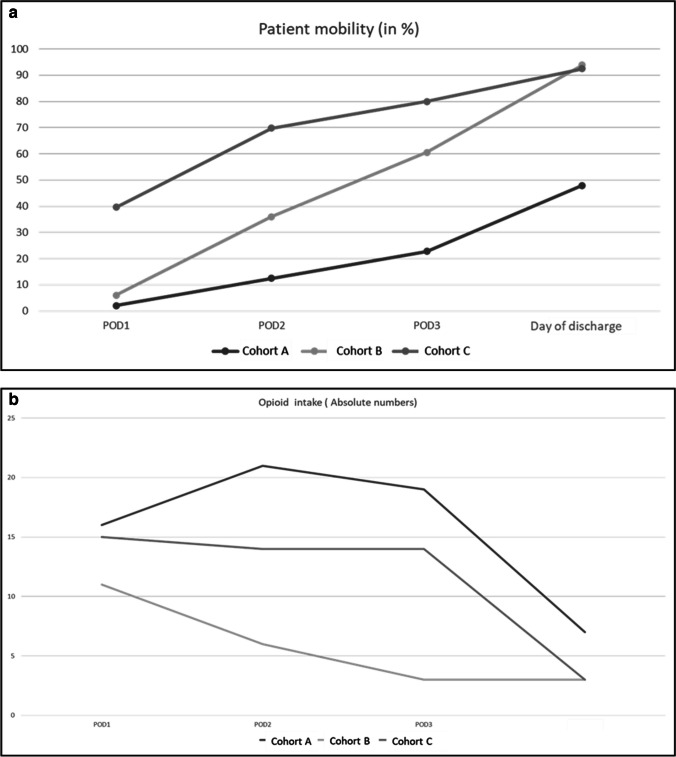
**a**, **b** Patient mobility on the surgical floor on postoperative days 1, 2, and 3 (POD1, POD2, and POD3) until day of discharge (%) (**a**), need for opioid medication on the surgical floor on postoperative days 1, 2, and 3 (POD1, POD2, and POD3) until day of discharge (in absolute numbers) (**b**)

Pain levels were evaluated using the VAS score as can be seen in Fig. 1. On POD1, the median VAS score in cohort A was 2.0, in cohort B 2.0, and in cohort C 4.0. The lowest pain levels were reported in cohort B on POD2 at 0.0. The median was 1.0 in cohort A and 3.0 in cohort C. On POD3, cohort A and cohort B again had the lowest score at 0.0, and cohort C was the highest at 2.0, with no significant difference between the three groups as shown in Fig. 2.

Regarding patient mobility, the cohorts showed a significant difference between the pre-ERAS® cohort (A) and the two ERAS® cohorts (B + C) (*p* < 0.001). Patient mobility was increased through the ERAS® protocol. On POD1, about 43% (*n* = 21) of patients in cohort A are mobile in the room or in the hallway, whereas in cohorts B and C, 85% (*n* = 43 and *n* = 48) of patients are mobile in the room or in the hallway. From POD2 to the day of discharge, the ERAS® cohorts showed significantly better mobility scores than the pre-ERAS® cohort (*p* < 0.001).

### PostopQRS™

Postoperative recovery was evaluated by the PostopQRS™ software. This was only performed in cohort C, as the tool cannot be retrospectively applied. On the day of discharge, 90% of patients scored the same as preoperatively regarding the capability of performing daily activities independently. The same result was achieved regarding cognitive abilities, where patients scored at 90% compared to before surgery. Emotional well-being and recovery were also described in 90% of the cases at the time of discharge. Regarding pain, 50% of the patients were fully recovered on the day of discharge, comparable to the findings in our study regarding pain levels and need for medication.

## Discussion and conclusion

Concluding from our data, patients who are treated following the ERAS® guidelines seem to profit from improved early postoperative recovery after elective colorectal resection. Our monocentric study revealed faster mobilization, earlier bowel movement, or stoma output in the ERAS® cohorts. Additionally, there was a reduced need for opioid medication and faster resumption of oral food intake early postoperatively (POD1). Prior evaluation after the ERAS® implementation at our hospital showed a reduced length of hospital stay with comparable complication rates between cohorts. Moreover, the tolerance for solid food early postoperatively was increased [5]. This is consistent with current evidence.

As can be seen in the literature, intraoperative use of minimally invasive procedures such as laparoscopic or robotic approaches combined with locoregional anesthesia (TAP block) leads to less need for opioid medication and peridural anesthesia [6–8]. This can also be concluded from our data, where pain levels are equally low as in the pre-ERAS® cohort despite reduced opioid use with significantly better patient mobility in both ERAS® cohorts. The combination of minimally invasive surgery (MIS) and the application of the TAP block appears to be an ideal alternative to epidural anesthesia within the context of ERAS®. Although the TAP block has been known for a long time, epidural anesthesia via a catheter has traditionally been the standard in colorectal surgery. In our hospital, the two-stage laparoscopic transversus abdominis plane block (L-TAPB) using bupivacaine at both the beginning and end of surgery is utilized as the standard. It has been demonstrated to be superior to epidural catheter anesthesia and a single-shot TAP block, with reduced requirements for oral and intravenous opioid intake early postoperatively when applied correctly [9]. The technique involves performing the bilateral L-TAPB twice during the procedure under laparoscopic visual control. Visual control is essential to ensure the correct layer is targeted, which is indicated by a slow and distinct bulge forming between the muscle layers during the L-TAPB application. A 21-gauge needle is used, and a total of 266 mg of bupivacaine (60 ml) diluted in a 2:1 ratio with 0.9% sodium chloride (30 ml) is administered [9].

It was proven that the early removal of a nasogastric tube as a part of the ERAS® program leads to both a quicker onset of bowel function and a reduction in the risk of pulmonary infections due to aspiration, as well as PONV [10]. Furthermore, early oral food intake facilitates a faster restoration of bowel function without an increased complication rate postoperatively [11]. Comparable studies show an average of 2.7 days to the first solid meal intake in the ERAS® cohort, compared to 4.7 days in the pre-ERAS® cohort (*p* < 0.001), which is consistent with our data [7].

The increased PONV rate in cohort C ultimately remains unclear but could also be due to more stringent data collection due to prospective data acquisition and thus correspond to a detection bias.

Süsstrunk et al. demonstrated that, even in a specialized center for colorectal surgery, adherence to the ERAS® protocol led to a reduction in non-surgical complications compared to standard care (*p* = 0.02). This was significant even though MIS and modern anesthesia care had already been standard practice in the standard care group. The mean length of stay (LOS) and mean costs per case were significantly lower in the ERAS® group compared to the standard care group (9.2 ± 5.6 days versus 12.7 ± 7.4 days, *p* < 0.01; costs 33,727 ± 15,883 USD versus 40,309 ± 29,738 USD, *p* < 0.01) [12].

As PostopQRS™ is a relatively new tool for the evaluation of patient recovery, comparable data is very limited. There are no studies comparing recovery after colorectal resection. As patients in our study scored at 90% recovery in all areas (capability of performing daily activities independently, emotional well-being, cognitive abilities) besides pain (50%), we suppose that a rapid recovery was achieved. Lee et al. [8] utilized the PostopQRS™ to compare recovery in 80 patients after laparoscopic gastrectomy. In their monocentric study, patients were surveyed and the PostopQRS™ questionnaire was administered 1 h, 1 day, and 6 days after surgery at Chonnam National University Hospital in South Korea. Complete recovery in terms of pain scores, depending on the cohort, was 22% and 23% on day 6 after surgery. In terms of cognitive recovery, patients in Lee’s study showed recovery rates of 83% and 84% on day 6 after surgery. Emotional recovery was around 87% on day 6 after surgery [8]. Additionally, data on PostopQRS™ after colonoscopy are available. These data are only partially comparable to colorectal surgery due to the minimal invasiveness of colonoscopy. However, even after colonoscopy, only 80% of patients were fully cognitively recovered on POD3. In this regard, the value in our study, with almost 80% of patients recovered on POD3 after colorectal resection, is likely to be considered satisfactory [13].

This study is a monocentric cohort study with a limited number of cases. Consequently, it is not randomized, which naturally constitutes a limitation and increases the risk of bias, especially selection bias. Due to the study design as a cohort study, data may be incompletely documented in the retrospective analysis. However, the strengths of the study include the high data quality of the prospectively collected data, the multifactorial mapping of the individual components of recovery and the innovative use of the PostopQRS™, a new measurement instrument for postoperative recovery. In terms of “implementation science,” this study highlights the important effects of the implementation of a colorectal ERAS® protocol.

## Supplementary Information

Below is the link to the electronic supplementary material.

Supplementary file1 (PDF 261 kb)

## Data Availability

The data that support the findings of this study are available upon request from the corresponding author.
