# Supplementary material for: Comparison of early postoperative recovery in patients undergoing elective colorectal surgery before and after ERAS® implementation—a single center three-armed cohort study
Source: Int J Colorectal Dis. 2024 Dec 2;39(1):194. doi: 10.1007/s00384-024-04770-0 (PMC11611963; doi:10.1007/s00384-024-04770-0)
Supplement: Supplementary file 1 — Supplementary file1 (PDF 261 kb) [file 384_2024_4770_MOESM1_ESM.pdf]

Compliance of ERAS® cohorts (B and C)

Cohort B (1 October 2020 – 31 July 2021)

Cohort C (1 August 2021 – 28 February 2022)

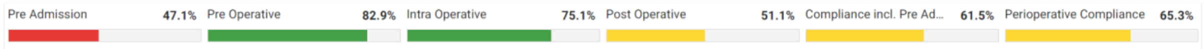

Fig. 1a: overall compliance during the various perioperative stages in Cohort B

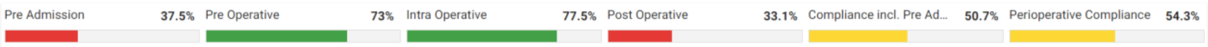

Fig. 1b: overall compliance during the various perioperative stages in Cohort C

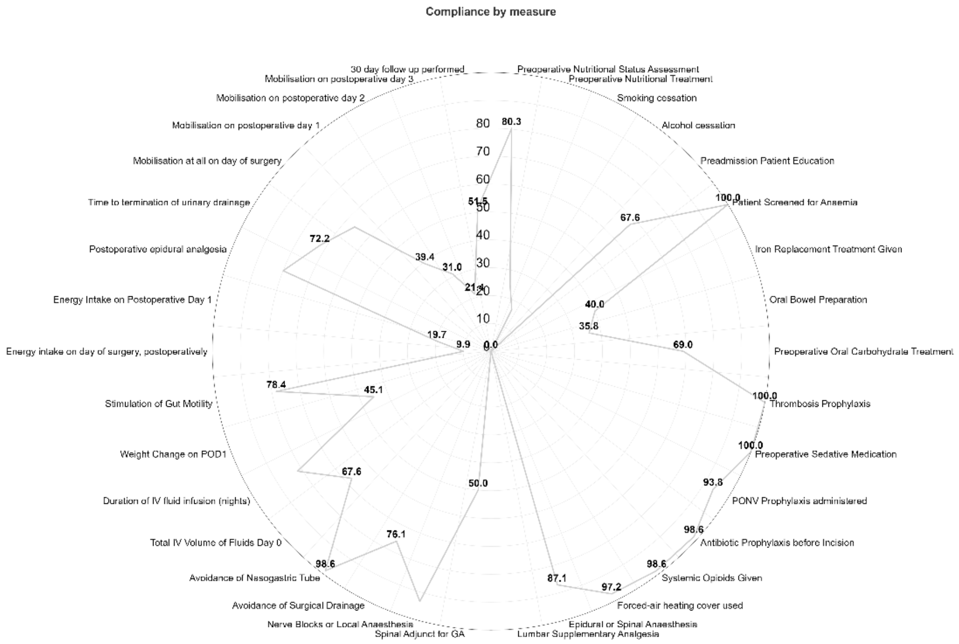

Fig. 2a: Compliance by measure - radar chart (Cohort B)

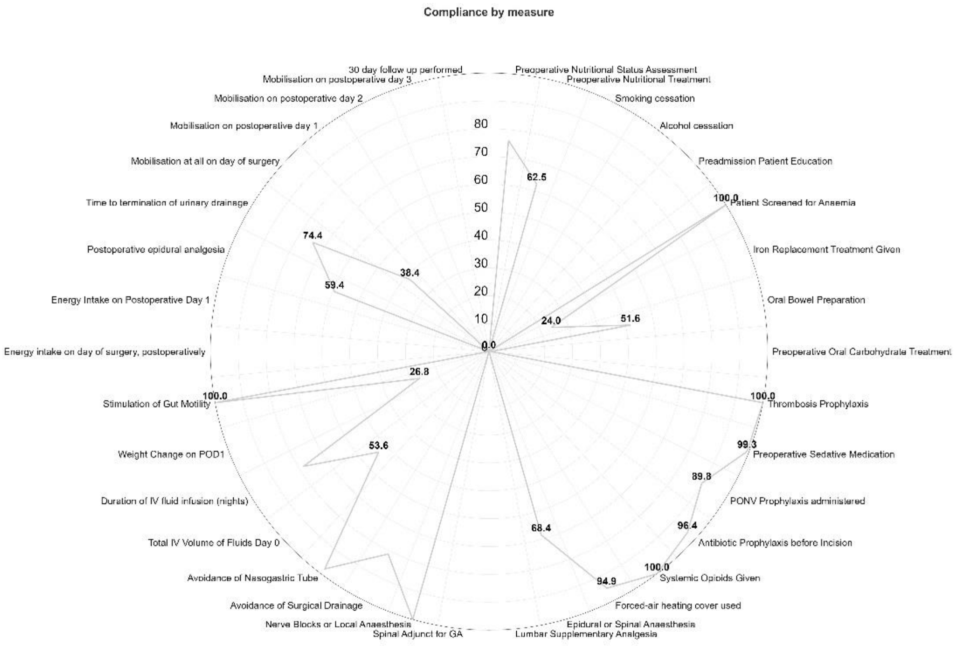

Fig. 2b: Compliance by measure - radar chart (Cohort C)
